# Regulation of *Aegilops tauschii* Coss Tiller Bud Growth by Plant Density: Transcriptomic, Physiological and Phytohormonal Responses

**DOI:** 10.3389/fpls.2020.01166

**Published:** 2020-07-29

**Authors:** Haiyan Yu, Hailan Cui, Jingchao Chen, Xiangju Li

**Affiliations:** Key Laboratory of Weed Biology and Management, Institute of Plant Protection, Chinese Academy of Agricultural Sciences, Beijing, China

**Keywords:** *Aegilops tauschii* Coss, plant density, tiller bud, RNA-Seq, Photosynthesis, plant hormones

## Abstract

*Aegilops tauschii* Coss is one of the most hazardous weeds that severely infests wheat fields in China. The tillering ability of *Ae. tauschii* strongly affects the occurrence and spread by influencing its seed output. In this study, *Ae. tauschii* was sown at low plant density (LPD) and high plant density (HPD) to investigate the effect of plant density on tiller bud outgrowth and its potential regulators using RNA-Seq. Additionally, the chlorophyll content and photosynthesis, soluble sugar and phytohormone levels were also determined at different plant densities. The results showed that an increased plant density significantly inhibited the elongation of tiller buds in the axil of the first leaf at 15 days after planting, with 7.69 mm at LPD and 1.69 mm at HPD. A total of seven putative tiller-related genes were selected and validated using quantitative real-time PCR. Furthermore, chlorophyll levels, photosynthetic efficiency, and soluble sugar contents were distinctly inhibited by HPD in *Ae. tauschii*, which may be responsible for the restriction of tiller bud growth. In addition, differentially expressed genes (DEGs) were markedly enriched in indole-3-acetic acid (IAA), abscisic acid (ABA), and gibberellin metabolism and signaling. Accordingly, the levels of ABA and gibberellin A3 in *Ae. tauschii* were strikingly higher at HPD compared with those at LPD, yet the reverse tendency was observed for IAA. Undoubtedly, such results will be highly beneficial for illuminating the underlying regulators of the *Ae. tauschii* tillering response to plant density and may provide new ideas for the control of this weed in the future.

## Introduction

*Aegilops tauschii* Coss, which belongs to the Poaceae family, is a notorious annual weed that greatly affects wheat yield and quality (Zhang et al., 2007). The weed spreads in the main wheat production areas of China and competes with wheat for light, water, and resources (Yu and Li, 2017). Presently, effective control methods of *Ae. tauschii* are lacking due to its close genetic relationship with wheat (Fang et al., 2015). *Ae. tauschii* completely relies on its seed for reproduction and to expand to new habitats. For Poaceae, the seed output is largely dependent on the number of tillers (Lecarpentier et al., 2019). Therefore, the accelerated infection and spread of *Ae. tauschii* greatly attributed to its high reproductivity are closely associated with its strong tillering ability.

The occurrence of tillers can be divided into two steps: the establishment of a tiller bud located in the axil of each leaf and its elongation (Li et al., 2003). It is well known that environmental signals and genetic factors are implicated with the occurrence of tillers (Rameau et al., 2015). Plant density, as one of the important environmental factors, plays an instrumental role in tiller production. For example, in rice, an increase in plant density would inhibit the actual tillering ability of an individual plant (Clerget et al., 2016; Tian et al., 2017). A similar result of fewer tillers produced per plant under high plant density was also found in wheat (Otteson et al., 2007). Furthermore, at the early stage of bud development, increased plant density could arrest tiller bud elongation in sorghum (Kebrom et al., 2006). However, in *Ae. tauschii*, whether plant density affects its tiller bud outgrowth is still unclear. As for the mechanisms of tillering in response to plant density, previously published papers have shown that increased plant density could result in some changes in density-derived cues such as lower photosynthetically active radiation and a severely reduced R (red light):FR (far red light) ratio, which would adversely affect tiller occurrence (Kasperbauer, 1987; Simon and Lemaire, 1987; Evers et al., 2006; Kebrom et al., 2006). In *Arabidopsis thaliana*, it was proposed a putative model in which increased plant density would trigger a low R:FR ratio, resulting in inactivity of Phytochrome B (phyB) and a rapid accumulation of phytochrome-interacting factors (PIFs) proteins. These PIFs repressed the expression of *MicroRNA156* (*MIR156*), which activated their target genes, and ultimately led to smaller branches (Xie et al., 2017). Besides, it was shown that PIFs (PIF7, PIF4, and PIF5) in *A. thaliana* regulated the shade-induced response by controlling the biosynthesis and signaling of auxin (Hornitschek et al., 2012; Lin et al., 2012). The role of auxin in establishment of apical dominance and suppression of branching is well known (Rameau et al., 2015). Based on that, light is likely to control the branching through interacting with auxin. Reddy and Finlayson (2014) proposed that phyB supressed auxin signaling to promote braching in *A. thaliana*. Obviously, the tiller development response to plant density is a complex process associated with several metabolism and signaling pathways. However, the underlying regulatory network for this process is not well understood.

Currently, numerous genes participating in the regulation of branching or tillering have been identified in *Oryza sativa*, *A. thaliana, Pisum sativum* L*., and Solanum lycopersicum*. For example, *MONOCULM1* (*MOC1*), a homolog to *Arabidopsis LATERAL SUPPRESSOR* (*LAS*) and *S. lycopersicum Lateral suppressor* (*Ls*), was first isolated from rice, and it plays an important role in the formation of tiller bud (Schumacher et al., 1999; Greb et al., 2003; Li et al., 2003). *MONOCULM3* (*MOC3*), *FLORAL ORGAN NUMBER1* (*FON1*), and *TEOSINTE BRANCHED1* (*TB1*) were reported to participate in the adjustment of tiller bud elongation (Doebley et al., 1997; Takeda et al., 2003; Aguilar-Martínez et al., 2007; Shao et al., 2019). Additionally, *PIN-FORME1* (*PIN1*), *ISOPENTENYL TRANSFERASE* (*IPT*), *DWARF17* (*D17*)*/MORE AXILLARY GROWTH3* (*MAX3*)*/RAMOSUS5* (*RMS5*), *DWARF3* (*D3*)*/MORE AXILLARY GROWTH2* (*MAX2*)*/RAMOSUS4* (*RMS4*), *DWARF14* (*D14*), and *DWARF53* (*D53*) indirectly regulate the occurrence of tiller through participating in plant hormone biosynthesis, transport, and signaling (Stirnberg et al., 2002; Booker et al., 2004; Ishikawa et al., 2005; Xu et al., 2005; Johnson et al., 2006; Zou et al., 2006; Ferguson and Beveridge, 2009; Jiang et al., 2013; Zhou et al., 2013). Critically, in *Ae. tauschii* no records of tiller-related genes have been found in the available scientific literature to date. Presently, identification and characterization of tiller-related genes using RNA-Seq have made great progresses in some crops (Kebrom and Mullet, 2016; Luo et al., 2019; Zhao et al., 2019). For example, in sorghum, RNA-Seq was used to analyze the gene expression in wild-type and *phy-B* mutant tiller buds. A transcriptome analysis of bud growth at the early stage revealed that some dormancy-related genes such as *Dormancy-associated protein-like1* (*DRM1*) and *Grassy tillers1* (*GT1*), together with *Early nodulin93* (*ENOD93*), *type-A response regulator3/6/9* (*ARR3/6/9*), *Short hypocotyl2* (*SHY2*), *Cytokinin-responsive GATA transcription factor22* (*CGA1*), and *1-Aminocyclopropane-1-carboxylic acid oxidase gene* (*ACO*), were associated with the arrest of tiller buds in *phy-B*; the outgrowth of wild-type tiller bud was possibly correlated with genes encoding a SWEET transport and cell wall invertase (Kebrom and Mullet, 2016). Clearly, RNA-Seq is an effective tool for characterizing the genes responsible for the phenotype of plant.

In *Ae. tauschii*, very little information is currently available regarding the effect of plant density on the outgrowth of tiller buds and its potential regulators. Therefore, the objectives of this study were to (i) evaluate the effect of plant density on the tiller bud growth; (ii) identify putative tiller-related genes from transcriptome analysis, and validate them using quantitative real-time PCR (qPCR); (iii) ascertain metabolism and signaling pathways involved in the tillering response to plant density and (iv) assay some relevant physiological indices. Undoubtedly, such results will contribute to further understanding the underlying regulators of *Ae. tauschii* tillering response to plant density and may provide new insight into the integrated management of this weed species in the future.

## Materials and Methods

### Plant Materials and Growth Conditions

*Ae. tauschii* seeds were collected from Henan Province, China (35.95°N, 114.90°E), which is the largest wheat-producing province with a serious *Ae. tauschii* infestation. Seeds were sown on trays (1.1 m × 0.84 m) containing 10 cm-deep cells filled with soil, vermiculite, and organic fertilizer (3:1:1). All trays were put into a growth chamber under a 14 h light/10 h dark (20°C/15°C) photoperiod with 500 µmol m-^2^s^-1^ light intensity and 65% humidity. Plants were watered as necessary to maintain optimal moisture of the soil.

### Growth Parameters Measurement and Sample Preparation

To simulate low plant density (LPD) and high plant density (HPD), seedlings were grown at a rate of 300 seedlings m^-2^ and 3,000 seedlings m^-2^, respectively. Each treatment contained three trays, and the experiment was independently repeated three times. In the pre-experiment, the tiller bud in the axil of the first leaf could be seen visually until 11 days after planting (DAP11), and differences in length between LPD and HPD could be detected until 13 days after planting (DAP13); therefore, plant height, the width and length of the first leaf, leaf number, and the length of tiller buds in the axil of the first leaf from thirty *Ae. tauschii* seedlings at different plant densities were measured at 11, 13, and 15 days after planting (DAP15). Plant height was assayed as the height from the base of the shoot to the tip of the tallest leaf. The width of leaf was measured on the center of the first leaf. Meanwhile, the tiller buds located at the axil of the first leaf from thirty seedlings were collected at the same point for RNA-Seq. Given the difficulty of obtaining sufficient numbers of tiller buds for the measurement of soluble sugar and plant hormones, the leaf and 0.5 cm of the basal part of stem (BPS) were cut from twenty individual plants at DAP11, DAP13, and DAP15 for further analysis. Seedlings grown near the margin of the trays were discarded during sampling.

### RNA Extraction, Library Construction, and Sequencing

Eighteen samples from LPD and HPD at DAP11, DAP13, and DAP15 were used for RNA extraction. Total RNA was extracted with Trizol, and the quality and quantity were evaluated using Agilent 2100 (Agilent, Santa Clara, CA). Total RNA was enriched and fragmented before the synthesis of cDNA with a random primer. Subsequently, the purified cDNA was ligated to generate 3’ adenine base overhangs after reparation, followed by performance of adaptor ligation on each library. The cDNA library was enriched through PCR amplification and then sequenced with Illumina HiSeqTM 4000 (Illumina, San Diego, CA).

### Sequence Alignment and Differential Expression Analysis

The quality control was strictly conducted on raw data. High-quality clean reads were mapped against the *Ae. tauschii* reference genome (assembly Aet_ MR_1.0). The gene expression level was calculated as fragments per kilobase of exon model per million mapped reads (FPKM). The genes with |log_2_ (Fold change) | > 1 and *q* value (adjusted *p*-value) < 0.05 were regarded as differentially expressed genes (DEGs). Expression differences of genes were compared between the LPD and HPD at DAP11 (DAP11_High vs DAP11_Low), DAP13 (DAP13_High vs DAP13_Low), and DAP15 (DAP15_High vs DAP15_Low). A Gene Ontology (GO) enrichment analysis was performed on DEGs with GOseq R package^29^ based on Wallenius’ noncentral hyper-geometric distribution (Young et al., 2010). All DEGs were also mapped to the Kyoto Encyclopedia of Genes and Genomes (KEGG) pathway.

### Quantitative Real-Time PCR Analysis

RNA samples used in qPCR analysis were from RNA-seq analysis. All qPCR primers were designed using Oligo 7 software **(** **Table S1** **)**. A total of 800 nanogram RNA was used for synthesis of the first-cDNA according to the procedure of TransScript^®^ All in One Frist-Strand cDNA Synthes is SuperMix for qPCR (Transgene, Beijing, China). qPCR was conducted with Bestar^®^ SybrGreen qPCR Mastermix (DBI^®^ Bioscience, Ludwigshafen, German) in a 20 μl reaction volume containing 10 μl of qPCR Mastermix, 8 μl of ddH_2_O, 0.5 μl of forward and reverse primer, and 1 μl of cDNA. *AeTubulin* was regarded as reference gene to normalize the expression level of target genes. The method of 2^-△△CT^ was used for the calculation of the gene expression level.

### Chlorophyll Pigment Content Assay

Two hundred milligrams of fresh leaves were used to extract a chlorophyll pigment mixture with 95% alcohol at 4°C in the dark until the tissue became completely bleached. Then, the extraction solvent was centrifuged at 4°C and 10,000 rpm for 5 min. The OD values of the supernatant were assayed spectrophotometrically at 470, 649, and 665 nm. The concentrations of Chla (C_a_), Chlb (C_b_), and Chl(a+b) (C_(a+b)_) were calculated according to C_a_=13.95OD_665_ – 6.88OD_649_, C_b_=24.96OD_649_ – 7.32OD_665_, and C_(a+b)_=6.63OD_665_ + 18.08OD_649_, respectively.

### Photosynthesis Assay

The net photosynthesis rate (A) and the transpiration rate (E) were measured on the center of the first fully expanded leaf of *Ae. tauschii* at different plant densities with a Walz GFS-3000 portable photosynthesis system (Heinz Walz GmbH) at DAP11, DAP13, and DAP15. The measured area was fully exposed to growth chamber lighting. The gas flow and photon flux density were set at 750 µmol s^-1^ and 500 µmol m^-2^s^-1^, respectively, with the CO_2_ concentration ranging from 650 to 680 µmol mol^-1^. Additionally, chlorophyll fluorescence parameters were also determined on the center of the first fully expanded leaf of *Ae. tauschii* at different plant densities with a Dual PAM-100 system (Heinz Walz GmbH) at DAP11, DAP13, and DAP15. The maximum PSII photochemical efficiency (*Fv*/*Fm*), the actual PSII photochemical efficiency (Y(II)), the nonphotochemical quenching (*NPQ*), the photochemical quenching coefficient (*qL*) and the PSII electron transport efficiency (ETR) were assayed after the plants were kept in the dark for 30 min.

### Carbohydrate Contents Assay

Fructose, glucose, trehalose, and sucrose were extracted from leaves and BPS of *Ae. tauschii* in accordance with the manufacturers’ instructions for the Fructose Microplate Assay Kit, Glucose Microplate Assay Kit, Trehalose Microplate Assay Kit and Sucrose Microplate Assay Kit (Cohesion Biosciences, England), respectively. The OD values of fructose, glucose, trehalose and sucrose were determined at 480, 505, 540, and 480 nm, respectively.

### Plant Hormone Content Assay

Indole-3-acetic acid (IAA), abscisic acid (ABA) and gibberellin A3 (GA_3_) were extracted from BPS based on a previously published method (Šimura et al., 2018), with some modifications. Powered samples (25 mg) were extracted in 1 ml of precooled 50% acetonitrile in water (v/v) with an isotopically labeled internal standard mixture added. The samples were vortexed for 30 s and sonicated for 5 min in ice-water bath followed by homogenization for 4 min and sonication for 5 min. The homogenate and sonicate circle were repeated twice. Supernatant (950 μl) was further purified with SPE (1 cc per 30 mg; waters, USA) after centrifugation at 12,000 rpm and 4°C for 10 min. The SPE cartridges were washed with 1 ml of methanol, and then equilibrated with 1 ml of 50% acetonitrile in water (v/v). The ﬂow-through fraction was discarded after loading a sample, followed by rinsing with 1 ml of 60% acetonitrile in water (v/v). The purified samples were evaporated to dryness under a gentle stream of nitrogen, and then reconstituted in 100 μl of 10% acetonitrile in water (v/v). The reconstituted solution was centrifuged at 12,000 rpm and 4°C for 15 min and then analyzed using EXIONLC System ultra-performance liquid chromatography (Sciex) tandem SCIEX 6500 QTRAP+ triple quadrupole mass spectrometer (Sciex) equipped with an IonDrive Turbo V electrospray ionization (ESI) interface (UPLC-MS/MS). A Waters ACQUITY UPLC CSH C_18_ column (150 mm × 2.1 mm, 1.7 μm) was operated at a column temperature of 50°C and injection volume of 5 μL. The mobile phase A was 0.01% formic acid in water, and the mobile phase B was 0.01% formic acid in acetonitrile. Typical ion source parameters of curtain gas, 40 psi; IonSpray voltage, ± 4500 V; temperature, 475°C; ion source gas 1, 30 psi; and ion source gas 2, 30 psi were used in this analysis. The MRM parameters of IAA, ABA and GA_3_ are shown in **Table S2**.

### Statistical Analysis

The mean value and standard deviation of all experimental data were calculated based on three independent biological replications. SPSS 21.0 software was used to analyze the significant differences by t-test at the 5% probability level.

## Results

### Increased Plant Density Inhibited the Outgrowth of Tiller Bud

**Figure 1:**
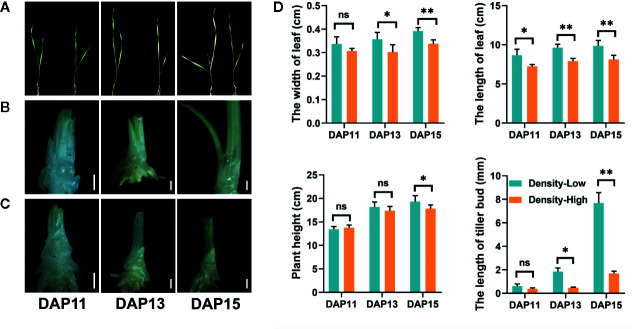
**(A)** Image of *Aegilops tauschii* Coss seedlings at low (left) and high (right) plant density at 11 (DAP11), 13 (DAP13), and 15 (DAP15) days after planting. **(B**–**C)** Growth status of tiller buds located in the axil of the first leaf at low **(B)** and high **(C)** plant density at DAP11, DAP13, and DAP15. The scale bar represents 1 mm. **(D)** The width and length of the first leaf, plant height, and length of tiller buds located in the axil of the first leaf at low and high plant density at DAP11, DAP13, and DAP15. Means and their SDs from three biological replicates are shown. Ns, no significance; *P < 0.05; **P < 0.01.

There were some differences in growth status of *Ae. tauschii* plants between LPD and HPD (**Figure 1**). The width and length of the first leaf were obviously inhibited by HPD, particularly at DAP13 and DAP15, indicating that *Ae. tauschii* leaves were bigger at LPD relative to HPD; at DAP15, plant density had significant effects on the plant height (*P* < 0.05) (**Figure 1D**). At DAP15, approximately 58% seedlings at LPD emerged the third leaves, yet two leaves per plant were observed at HPD during the whole experiment period. Additionally, plant density strikingly affected the outgrowth of tiller bud located in the axil of the first leaf in *Ae. tauschii*. At DAP11, there was no significant difference in tiller bud length between LPD and HPD. However, at DAP13 and DAP15, the LPD tiller buds were 4.0- and 4.6-fold longer than the HPD tiller buds, respectively (**Figures 1B–D**).

### Global Analysis of Transcriptome

**Figure 2:**
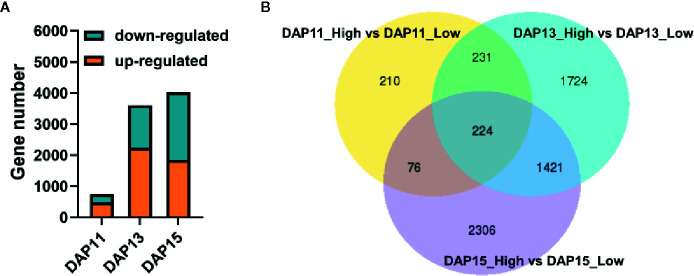
Differentially expressed genes (DEGs) of the *Aegilops tauschii* Coss tiller bud response to plant density. **(A)** The number of upregulated and downregulated DEGs at DAP11, DAP13, and DAP15. **(B)** Venn diagram of the three comparison groups of DEGs.

A total of 1062.5 million raw reads were obtained from 18 RNA libraries after RNA-Seq ranging from 46.16 million reads to 84.45 million reads. Among them, 96.60 to 97.56% per library were clean reads after quality control, and 94.21 to 96.13% of the clean reads were successfully mapped to the *Ae. tauschii* genome with a unique map ranging from 90.47 to 92.20% (**Table 1**). LPD treatment was selected as the control in the experiments. At DAP11, a total of 741 DEGs were detected in tiller bud, with 484 upregulated and 257 downregulated. A total of 3,600 DEGs were identified, of which 2,243 were upregulated, and 1,357 were downregulated at DAP13. At DAP15, 4,027 DEGs were noted, with 1,841 up-regulated genes and 2,186 down-regulated genes (**Figure 2A**). Besides, 455 DEGs detected at DAP11 were also noted at DPA13. DAP13 and DAP15 shared 1,645 DEGs, and DAP11 and DAP15 shared 300 DEGs (**Figure 2B**).

**Table 1:** Summary of RNA sequencing and mapping data with the *Ae. tauschii* genome (assembly Aet_ MR_1.0) as the reference*^a^*.

Sample	Total reads	Clean reads	GC (%)	Total mapped (%)	Uniquely mapped (%)	Multiple mapped (%)
DAP11_Low_1	62270760	60601904	55.55	95.75	91.48	4.27
DAP11_Low_2	52576474	51293608	55.98	95.83	91.72	4.11
DAP11_Low_3	72119880	70187068	55.62	95.80	91.65	4.16
DAP11_High_1	71599589	69695040	55.35	95.96	91.93	4.03
DAP11_High_2	84448045	81830156	55.84	95.89	91.59	4.30
DAP11_High_3	71004362	68789026	55.61	95.75	91.56	4.18
DAP13_Low_1	84105371	81859758	55.37	96.09	91.81	4.28
DAP13_Low_2	46155563	44793974	55.35	95.97	91.67	4.30
DAP13_Low_3	54763577	53022096	55.18	94.21	90.47	3.74
DAP13_High_1	50012238	48376838	55.43	96.01	92.19	3.82
DAP13_High_2	50694517	49133126	55.25	95.50	91.17	4.33
DAP13_High_3	54551046	52810868	55.09	95.67	91.41	4.26
DAP15_Low_1	51772070	50011820	54.80	95.63	91.42	4.21
DAP15_Low_2	47992256	46408512	54.83	95.76	92.04	3.72
DAP15_Low_3	53371766	51743928	54.80	96.04	91.87	4.17
DAP15_High_1	51046738	49423452	55.08	96.03	92.20	3.82
DAP15_High_2	53360273	51551360	55.16	95.78	91.49	4.29
DAP15_High_3	50677342	49390138	55.49	96.13	91.77	4.36

^a^Columns represent the number of raw reads, the number of clean reads, GC content and ratio of sequences mapped to the genome. The numerals 1, 2, and 3 represent the different biological replications. DAP11_Low, DAP13_Low, and DAP15_Low: tiller buds collected from low plant density at 11, 13, and 15 days after planting, respectively; DAP11_High, DAP13_High, and DAP15_High: tiller buds collected from high plant density at 11, 13, and 15 days after planting, respectively.

### GO and KEGG Enrichment Analysis

To gain insight into the DEGs functional categories, GO enrichment analysis was used to evaluate the specific functional role of all the DEGs. The “electron transport” and “translation” were the top GO terms in the “biological process” group. The “cytoplasm” and “macromolecular complex” were significantly enriched in “cellular component” group. The “structural molecular activity” and “oxidoreductase activity” categories were the most enriched GO terms in the “molecular function” group (**Figure S1**).

**Figure 3:**
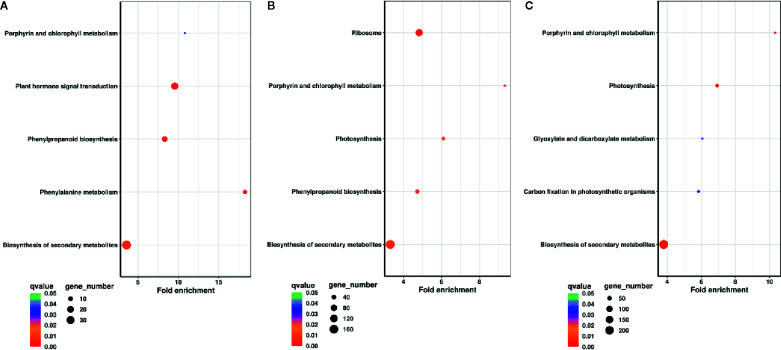
KEGG pathway enrichment analysis of DEGs at DAP11 **(A)**, DAP13 **(B)**, and DAP15 **(C)**. Fold enrichment in a pathway means the ratio of the number of observed DEGs and expected DEGs.

To understand the functional enrichment classification of DEGs, KEGG analysis was carried out. Biosynthesis of secondary metabolites, plant hormone signal transduction, phenylpropanoid biosynthesis, phenylalanine metabolism, and porphyrin and chlorophyll metabolism were significantly enriched pathways at DAP11 (*q* < 0.05) (**Figure 3A**). Ribosome, porphyrin and chlorophyll metabolism, photosynthesis, biosynthesis of secondary metabolites, and phenylpropanoid biosynthesis were the top enriched pathways at DAP13 (*q* < 0.05) (**Figure 3B**). Porphyrin and chlorophyll metabolism, photosynthesis, biosynthesis of secondary metabolites, carbon fixation in photosynthetic organisms, and glyoxylate and dicarboxylate metabolism were predominantly enriched pathways at DAP15 (*q* < 0.05) (**Figure 3C**). For upregulated DEGs, plant hormone signal transduction was a shared pathway markedly enriched in DAP11, DAP13, and DAP15 (*q* < 0.05). In terms of downregulated DEGs, three shared pathways were highly enriched in DAP13 and DAP15: porphyrin and chlorophyll metabolism, photosynthesis, and carbon fixation in photosynthetic organisms (*q* < 0.05). (**Figure S2**). In porphyrin and chlorophyll metabolism pathway, a total of 26 DEGs regulating the synthesis of chlorophyll pigments were determined. All of these genes were dramatically downregulated at HPD in comparison with LPD, particularly at DAP13 and DAP15. With the outgrowth of tiller buds, the decrease in gene expression was markedly enhanced (**Figure S3**). Besides, in the pathways of photosynthesis and carbon fixation in photosynthetic organisms, most DEGs involved in photosynthetic electron transport and the Calvin cycle exhibited much lower expression at HPD than at LPD at DAP13 and DAP15, whereas at DAP11 their expression was nearly similar (**Figure S3**).

### Candidate Tiller-Related Gene Selection and Validation

To obtain more information, candidate genes were selected from DEGs at DPA11, and shared DEGs at DAP13 and DAP15. Furthermore, among the above selected genes, DEGs were identified according to the knowledge of the molecular mechanisms regulating shoot branching published previously (Shimizu-Sato and Mori, 2001; Rameau et al., 2015; Wang and Wang, 2015; Kebrom and Mullet, 2016; Wang B. et al., 2018; Luo et al., 2019). MIR156-SPL (*SQUAMOSA PROMOTER BINDING PROTEIN-LIKE*) regulatory module is well believed to participate in branch and tiller development in plant species (Wang and Wang, 2015). A DEG belonging to *SPL* family (*LOC109766141*, *SPL10*) was selected from transcriptomic analysis. It was reported that changed light quality (R:FR) associated with neighboring plants influenced plant branching and tillering (Rameau et al., 2015). Far-red elongated hypocotyl1 (*LOC109778687*, FHY1) crucial for FR light signaling was considered as a putative tiller-related gene. Multiple researches uncovered that cell cycle genes were involved in arrest and maintenance of bud growth through the regulation of cell division (Kebrom and Mullet, 2016; Luo et al., 2019). A DEG annotated to *Cyclin-dependent protein kinases* family (*LOC109732743*, *CDK2*) was identified. Besides, axillary buds formed from axillary meristem is initially dormant and its later growth requires the releasing from dormancy (Shimizu-Sato and Mori, 2001; Wang B. et al., 2018). Three DEGs annotated to *DRM* family (*LOC109751939*, *LOC109772458* and *LOC109759890*) and a DEG encoding *ABSCISIC ACID 8’-HYDROXYLASE3* (*LOC109763237*, *ABA8ox3*) were obtained. Based on these described criteria, a total of seven DEGs were selected as putative tiller-related genes which may regulate the outgrowth of tiller bud in *Ae. tauschii* (**Table 2**). To verify this result, the expression pattern of the seven candidate genes were validated with qPCR, which was strongly consistent with the RNA-Seq data (**Table 2**).

**Table 2:** qPCR validation of putative tiller-related differentially expressed genes identified in RNA-Seq*^a^*.

GeneBank accession number	log_2_[fold change (High plant density/Low plant density)]								Gene description
	DAP11			DAP13			DAP15		
	RNA-Seq	qPCR		RNA-Seq	qPCR		RNA-Seq	qPCR	
*LOC109751939*	—	4.25*		5.27**	7.35**		7.03**	6.06**	Dormancy-associated protein1
*LOC109772458*	—	1.04		2.93*	3.30**		3.09**	3.54**	Dormancy-associated protein homolog3
*LOC109759890*	—	0.23		1.34**	3.20*		3.19**	3.27*	Dormancy-associated protein homolog3
*LOC109778687*	—	0.17		1.40**	3.07**		1.42**	2.31*	Far-red elongated hypocotyl1
*LOC109763237*	-1.39**	-1.28*		-2.06**	-1.57*		-1.69**	-0.97*	Abscisic acid 8’-hydroxylase3
*LOC109732743*	-3.01**	-2.60**		-4.69**	-2.37**		—	-0.10	Cyclin-dependent kinaseF-2
*LOC109766141*	—	-1.20*		-1.17**	-2.44*		-1.73**	-0.32	Squamosa promoter binding protein-like10

^a^DAP11, DAP13, and DAP15 represent at 11, 13, and 15 days after planting, respectively. — represents the genes were not considered as differentially expressed genes. * and ** represent the gene expression is significantly different between high plant density and low plant density at P < 0.05 and P < 0.01 respectively.

### High Plant Density Resulted in Weak Photosynthesis

**Figure 4:**
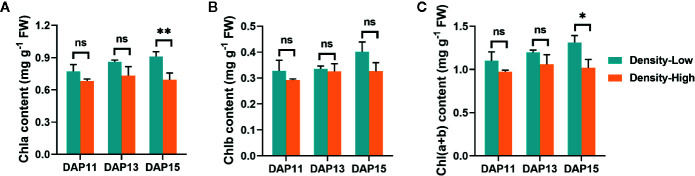
Chlorophyll contents detected in *Aegilops tauschii* Coss leaves at DAP11, DAP13 and DAP15. **(A)** Chlorophyll a, Chla; **(B)** Chlorophyll b, Chlb; **(C)** Chlorophyll a+b, Chl(a+b). Means and their SDs from three biological replicates are shown. Ns, no significance; *, *P* < 0.05; **, *P* < 0.01.

It is well established that increased plant density results in changed light quality and quantity due to competition for light form neighboring plants (Holmes and Smith, 1977; Simon and Lemaire, 1987; Rameau et al., 2015). Thus, the photosynthetic capability of *Ae. tauschii* may be influenced by enhanced plant density. To confirm this speculation, chlorophyll contents in leaves and photosynthesis of *Ae. tauschii* were assayed at different plant densities. At LPD, the chlorophyll contents [Chla, Chlb, and Chl(a+b)] gradually increased with the elongation of the tiller bud, whereas it was relatively steady at HPD. Additionally, the Chla contents at LPD were always higher than at HPD, especially at DAP15 (*P* < 0.01). Similar phenomena were detected for Chlb and Chl(a+b) as well (**Figure 4**).

**Figure 5:**
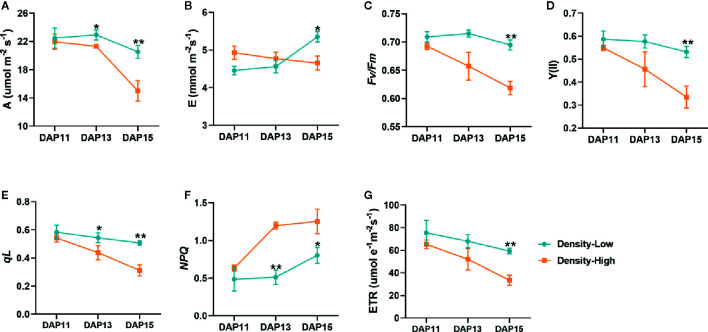
Photosynthesis and chlorophyll fluorescence parameters determined on the first expanded leaves of *Aegilops tauschii* Coss at DAP11, DAP13, and DAP15. **(A)** Net photosynthesis rate, A; **(B)** the transpiration rate, E; **(C)** the maximum PSII photochemical efficiency, *Fv/Fm*; **(D)** the actual PSII photochemical efficiency, (Y(II)); **(E)** the photochemical quenching coefficient, *qL*; **(F)** the nonphotochemical quenching, *NPQ*; **(G)** the PSII electron transport efficiency, ETR. Means and their SDs from three biological replicates are shown. *P < 0.05; **P < 0.01.

As for photosynthetic ability of *Ae. tauschii*, there were significant differences between HPD and LPD. Specifically, LPD showed a 1.1- and 1.4-fold higher net photosynthesis rate (A) than HPD at DAP13 (*P* < 0.05) and DAP15 (*P* < 0.01), respectively, yet it displayed a similar rate at DAP11 **(** **Figure 5A** **)**. At DAP15, the transpiration rate (E) was distinctly decreased at HPD compared with LPD (*P* < 0.05) **(** **Figure 5B** **)**. Additionally, in comparison with LPD, *Fv*/*Fm* and Y(II) were significantly lower at HPD, particularly at DAP13 and DAP15 **(** **Figures 5C, D** **)**, indicating that the ability of PSII to convert solar energy was largely inhibited by HPD. The change in dynamics of *qL* and ETR were almost similar with *Fv*/*Fm* and Y(II) **(** **Figures 5E, G** **)**, implying that PSII of *Ae. tauschii* at HPD absorbed little light utilized in photosynthetic electron transport and exhibited a lower electron transport rate. However, a notable increase in *NPQ* was detected in the plant at HPD **(** **Figure 5F** **)**, demonstrating increased thermal energy dissipation at HPD. Taken together, photosynthesis of *Ae. tauschii* was dramatically restricted at HPD (**Figure 5**). Based on that, we speculated that weaker photosynthesis of *Ae. tauschii* at HPD cannot provide sufficient energy for tiller bud development.

### Soluble Sugar Content Responded to High Plant Density

**Figure 6:**
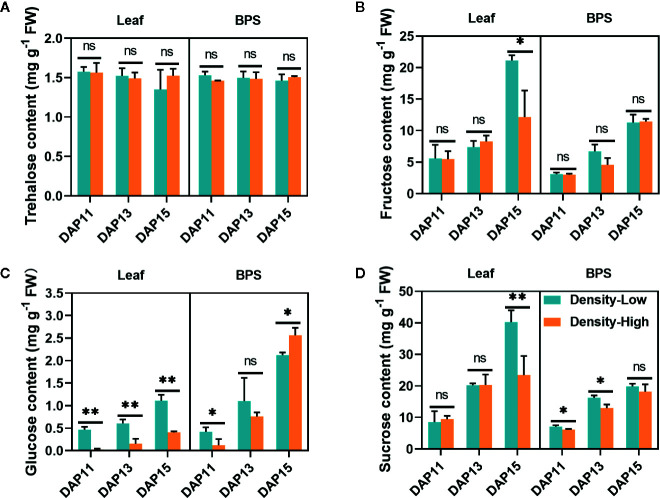
Trehalose **(A)**, fructose **(B)**, glucose **(C)**, and sucrose **(D)** contents detected in *Aegilops tauschii* Coss leaves and the 0.5 cm base part of the stem (BPS) at DAP11, DAP13, and DAP15. Means and their SDs from three biological replicates are shown. Ns, no significance; *P < 0.05; **P < 0.01.

To confirm the above hypothesis, the carbohydrate contents were assayed in leaves and BPS under different plant densities. There was no significant difference in trehalose content between LPD and HPD during the whole experimental period (**Figure 6A**), yet the fructose level in the leaves was 1.7-fold higher at LPD compared than at HPD at DAP15 (*P* < 0.05) (**Figure 6B**). The glucose content in the leaves and BPS continuously increased with the elongation of tiller buds. In contrast with LPD, HPD led to stronger reduction in glucose contents in leaves (*P* < 0.01), which was consistent with the lower photosynthesis efficiency at HPD. A similar trend was detected in BPS except for at DAP15 (**Figure 6C**). Sucrose was predominant among the four examined soluble sugars. The sucrose content in leaves and BPS was gradually enhanced with the development of tiller buds. In leaves, its levels were approximately similar between LPD and HPD, whereas at DAP15 more sucrose was accumulated at LPD (*P* < 0.01). In BPS, sucrose exhibited prominently higher levels at LPD than at HPD except for no significant difference at DAP 15 (**Figure 6D**).

### Phytohormones Affected Tiller Bud Growth

**Figure 7:**
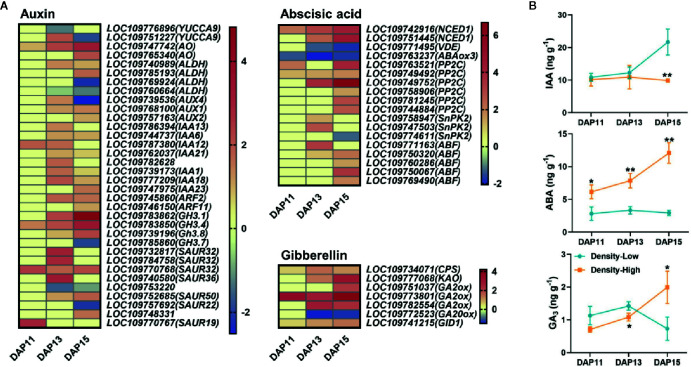
**(A)** The expression levels of DEGs involved in the biosynthesis and signaling of auxin, abscisic acid, and gibberellin based on log_2_(fold change) with low plant density treatment as the control at DAP11, DAP13, and DAP15. Each column represents different times, and each row represents one gene. **(B)** Contents of indole-3-acetic acid (IAA), abscisic acid (ABA), and gibberellin A3 (GA_3_) detected in the 0.5 cm base part of the stem in *Aegilops tauschii* Coss at DAP11, DAP13, and DAP15. Means and their SDs from three biological replicates are shown. Ns, no significance; *P < 0.05; **P < 0.01.

A total of 34 DEGs participated in the biosynthesis and signaling of auxin. At DAP13, many DEGs at HPD were upregulated in contrast with LPD. *NINE-CIS-EPOXYCAROTENOID DIOXYGENASE1* (*NCED1*), a gene responsible for the biosynthesis of ABA, exhibited a visibly higher expression level at HPD, while the opposite phenomenon was determined for the ABA degradation-related gene *ABA8ox3*. In addition, 14 DEGs related to ABA signaling displayed a distinctly higher expression level at HPD. DEGs associated with the biosynthesis and signaling of gibberellin also exhibited increased expression level at HPD (**Figure 7A**). To verify this finding, the levels of IAA, ABA, and GA_3_ in BPS of *Ae. tauschii* were assayed at DPA11, DAP13, and DAP15. There were relatively similar levels of IAA between HPD and LPD at DAP11 and DAP13, whereas at DAP15 the IAA contents were prominently increased at LPD, 2.2-fold higher than at HPD (*P* < 0.01). At HPD, ABA contents was sharply increased with the development of tiller bud, yet at LPD, it exhibited approximately similar levels. Compared with LPD, higher levels of ABA were determined at HPD during the whole experimental period, largely supporting the relevant gene expression in RNA-Seq. At HPD, GA_3_ was gradually accumulated with the tiller bud growth; until DAP15 its significantly higher level was found in BPS of *Ae. tauschii* (*P* < 0.05) (**Figure 7B**).

## Discussion

Plant density, as an important environmental factor, imposed a striking inhibitory effect on the outgrowth of tiller bud for *Ae. tauschii* (**Figure 1**). Increased plant density has been reported to inhibit the tiller bud elongation of sorghum (Kebrom et al., 2006), which greatly supported for our findings. Subsequent growth of tiller bud is one of important steps for the occurrence of tiller (Li et al., 2003). Undoubtedly, the arrest of the tiller bud growth in *Ae. tauschii* will result in fewer tiller numbers. Weak tillering of *Ae. tauschii* is directly responsible for decreased seed production (Cai et al., 2018), which may delay its establishment and distribution across wheat fields.

Seven putative tiller-related genes were selected from transcriptome analysis in *Ae. tauschii*, and their expression patterns were favored by qPCR validation, indicating that they were presumably involved in tiller bud outgrowth. Among seven putative tiller-related genes, three *DRM* genes (*LOC109751939*, *LOC109772458*, and *LOC109759890*) showed dramatically weaker expression at LPD in comparison with HPD. *DRM* has been reported to be tightly associated with dormancy in several plant species and considered as a molecular marker of dormancy (Stafstrom et al., 1998; Kebrom et al., 2006; Rae et al., 2013). Generally, axillary buds formed from axillary meristem is initially dormant or inhibited before being activated to develop into branch or tiller (Wang B. et al., 2018). Given that, it is speculated that enhanced expression levels of *DRM* genes at HPD is related with the dormancy of *Ae. tauschii* tiller bud. The correlations between *DRM* and tiller bud dormancy were also found in tall fescue and sorghum (Kebrom and Mullet, 2016; Zhuang et al., 2019), highly supporting for our speculations. However, Finlayson (2007) found that there was no correlation between *DRM1* expression and dormancy. Thus, the specific relationships between *DRM* and tiller bud outgrowth in *Ae. tauschii* need to be investigated furtherly. The expression of FHY1 (*LOC109778687*) was obviously enhanced in *Ae. tauschii* tiller bud at HPD. It is well believed that increased FR light has an inhibitory effect on the elongation of lateral bud (Xie et al., 2017; Wang B. et al., 2018). FHY1 is crucial for the Phytochrome A-mediated FR light signaling (Desnos et al., 2001; Chen et al., 2012). Therefore, FHY1 may be related with tiller bud development in *Ae. tauschii* through participating in signal transduction in perception of R:FR, whereas this hypothesis requires empirical testing for its validation. *SPL* gene family was found to potentially control crop important agronomic traits and improve yield (Jiao et al., 2010; Wang et al., 2012; Si et al., 2016). For example, *IDEAL PLANT ARCHITECTURE1*, encoding *OsSPL14*, participated in the regulation of the occurrence of tillers in rice (Jiao et al., 2010). In this study, *SPL10* (*LOC109766141*), belonging to *SPL* gene family, differentially expressed between HPD and LPD in *Ae. tauschii*. Marked differences in *ABA8ox3* (*LOC109763237*) expression were observed between HPD and LPD during *Ae. tauschii* tiller bud outgrowth. *ABA8ox3*, encoding an enzyme catalyzing ABA degradation, is a key determinant of the ABA level that is related with bud dormancy (Saito et al., 2004). Wang H. et al. (2018) found that transgenic lines of barley with down-regulation of *ABA8ox1* and *ABA8ox3* mediated by RNAi exhibited increased tiller formation. Therefore, it is likely that *ABA8ox3* can regulate the tiller bud elongation in *Ae. tauschii*. Cell cycle progression is found to be related with bud dormancy and growth (Shimizu and Mori, 1998). Differential expression patterns of cell cycle genes were detected in growth-arrested tiller bud in sorghum (Kebrom and Mullet, 2016). Luo et al. (2019) found that some genes related to cell cycle were enhanced in tiller bud of *dwarf10*, yet suppressed in dormant buds. *Tillering and Dwarf1* (*TAD1*), a component of the cell cycle machinery, can interact with *anaphase-promoting complex10* (*APC10*) to specifically recruit *MOC1* and then degrade *MOC1* to control rice tillering (Xu et al., 2012). In present study, *CDK2* (*LOC109732743*), belonging to *CDK* family which is implicated with the regulation of cell cycle progression (Polyn et al., 2015), had different expression levels between HPD and LPD. In the future, however, more investigative work is indispensable to elucidate the actual functions of these genes in *Ae. tauschii*. Moreover, these candidate tiller-related genes probably provide molecular targets for the development of RNAi herbicide to decrease *Ae. tauschii* tillering in the future.

Interestingly, in RNA-Seq, DEGs were significantly enriched in porphyrin and chlorophyll metabolism, photosynthesis and carbon fixation in photosynthetic organism pathways, and lower expressions of genes manipulating the biosynthesis of chlorophyll and photosynthesis were detected in *Ae. tauschii* tiller buds at HPD. However, tiller bud is located in the axil of each leaf and is shielded by the overlying leaf sheath. It is unlikely that it can conduct much in the way of meaningful photosynthesis. The reasons explaining this interesting phenomenon are worth further investigating in the future.

Compared with LPD, decreased chlorophyll levels were detected in *Ae. tauschii* leaves at HPD. Previous studies have described that light is essential for the biosynthesis of chlorophyll and that plant density observably influenced the contents of chlorophyll (Hashemi-Dezfouli and Herbert, 1992; Aminifard et al., 2010), which was strongly concordant with the observations in this study. Chlorophyll is crucial for photosynthesis through absorbing and transferring light. As expected, weaker photosynthetic capability was observed in *Ae. tauschii* leaves at HPD. Photosynthesis provides organic matter for the growth and development of plants (Nelson, 2011). Accordingly, *Ae. tauschii* generated lower levels of soluble sugar in leaves at HPD than at LPD. Carbohydrates are transported to other plant organs after they are produced in the leaves to supply energy for normal development. Sucrose is the main soluble sugar transported to other parts of the plants (Khayat and Zieslin, 1987). The sucrose levels in *Ae. tauschii* BPS were reduced at HPD in contrast with LPD during the outgrowth of its tiller buds, suggesting low photosynthetic ability and decreased sucrose transport from leaves to BPS in the plants at HPD. Given that lower soluble sugars have distinctly inhibitory roles in normal plant growth, weaker photosynthesis of *Ae. tauschii* at HPD cannot provide sufficient energy for the normal elongation of tiller buds, revealing that the outgrowth of tiller bud requires sufficient energy. A consistent phenomenon has been found in the wheat mutant *duc* with fewer tillers, and the restriction of its tillering was due to the lower photosynthesis rate of leaves and insufficient energy for the outgrowth of its tiller buds (An et al., 2019). Koumoto et al. (2013) proposed that the supply of sucrose might be a vital factor for the development of tiller bud of the rice monoculm mutation *moc2*, largely supporting the findings in this research. Interestingly, LPD tiller buds were obviously longer than HPD tiller bud with approximate 4-fold longer at LPD, whereas the differences in sucrose contents among different plant densities were minor with about 20% sucrose reduced at HPD, suggesting that other underlying factors were involved in tiller bud growth response to plant density as well.

Many DEGs involved in the biosynthesis and signaling of auxin, ABA and GA_3_ had higher expression levels at HPD, revealing that the metabolism of plant hormones is disturbed by HPD. Inconsistently, decreased IAA levels were found at HPD compared with LPD. Auxin cannot enter tiller buds (Prasad et al., 1993; Booker et al., 2003), and the auxin transport canalization hypothesis proposes that the establishment of auxin flow from the axillary bud into the main stem is essential for the sustained elongation of bud growth (Bennett et al., 2006; Prusinkiewicz et al., 2009). Therefore, we speculated that increased IAA levels in BPS at LPD were due to auxin transport from the tiller bud to the main stem. Consistently, increased plant density stimulated the accumulation of ABA and GA_3_ in BPS of *Ae. tauschii*, where tiller bud development was inhibited, suggesting they negatively regulated tiller bud outgrowth. ABA can induce the dormancy of seeds and buds (Shu et al., 2016; Nguyen and Emery, 2017). Therefore, higher levels of ABA in BPS at HPD might prevent the *Ae. tauschii* tiller buds releasing from dormancy, resulting in the arrest of tiller bud elongation. ABA has also been implicated in the regulation of SL-mediated axillary bud dormancy in rice (Luo et al., 2019). Wang H. et al. (2018) found that ABA might affect the tillering in barley through regulating SL biosynthesis. ABA biosynthesis mutants exhibited inadequate arrest in axillary bud growth in low R:FR (Reddy et al., 2013). Besides, the differences in ABA accumulation between LPD and HPD were large and occurred prior to the observed differences in tiller bud growth. Previously published paper described that there was a sustained decline of ABA contents and signaling at high R:FR, prior to increased axillary bud outgrowth, similar with the present findings to some extent (Holalu and Finlayson, 2017). As for GA, it has been found that it may inhibit bud growth (Rameau et al., 2015), which was greatly similar with the observations in this study. Collectively, the restriction of tiller bud growth at HPD may be caused by the abnormal plant hormone metabolism in *Ae. tauschii*. Given this hypothesis, external application of exogenous hormones or herbicides acting to disturb plant hormonal balance may be a good method for inhibiting *Ae. tauschii* tillering.

## Conclusion

In conclusion, increased plant density distinctly restricted the development of *Ae. tauschii* tiller bud. Seven putative tiller-related genes were identified and validated in *Ae. tauschii*. Furthermore, increased plant density might inhibit the outgrowth of *Ae. tauschii* tiller buds through decreasing photosynthesis and disturbing plant hormone metabolism. Based on these results, we can develop some RNAi herbicides targeted at silencing tiller-related genes to decrease *Ae. tauschii* tillering. Additionally, external spraying of exogenous hormones or herbicides acting to disturb photosynthesis or plant hormones balance may be a good choice to inhibit *Ae. tauschii* tillering and ultimately decrease its seed output.

## Data Availability Statement

The raw sequence data has been deposited in the NCBI Sequence Read Archive (SRA) database with accession number of SRP256104.

## Author Contributions

XL conceived the study. HY conducted the experiments and participated in the manuscript writing. HC participated in the seed collection. JC provided writing assistances.

## Funding

This work was supported by the National Key Research and Development Program of China (2016YFD0300701).

## Conflict of Interest

The authors declare that the research was conducted in the absence of any commercial or financial relationships that could be construed as a potential conflict of interest.
