# Supplementary material for: Regulation of Aegilops tauschii Coss Tiller Bud Growth by Plant Density: Transcriptomic, Physiological and Phytohormonal Responses
Source: Front Plant Sci. 2020 Jul 29;11:1166. doi: 10.3389/fpls.2020.01166 (PMC7403227; doi:10.3389/fpls.2020.01166)
Supplement: Supplementary file 1 [file DataSheet_1.docx]

Supplementary Material


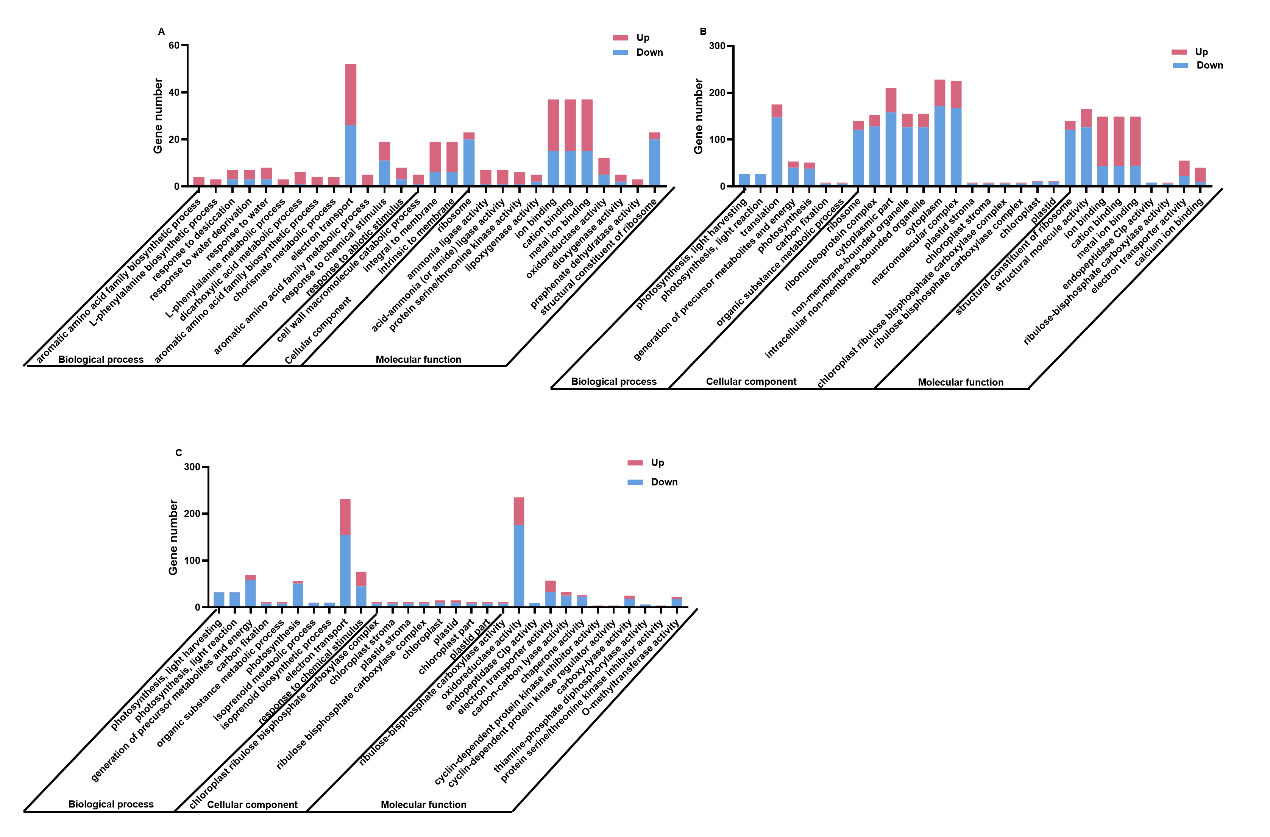


**Figure S1.** GO enrichment analysis of DEGs at DAP11 (A), DAP13 (B) and DAP15 (C).

**Figure S2.** KEGG pathway enrichment analysis of upregulated (A-C) and downregulated (D-F) DEGs at DAP11 (A and D), DAP13 (B and E) and DAP15 (C and F). Fold enrichment in a pathway means the ratio of the number of observed DEGs and expected DEGs.


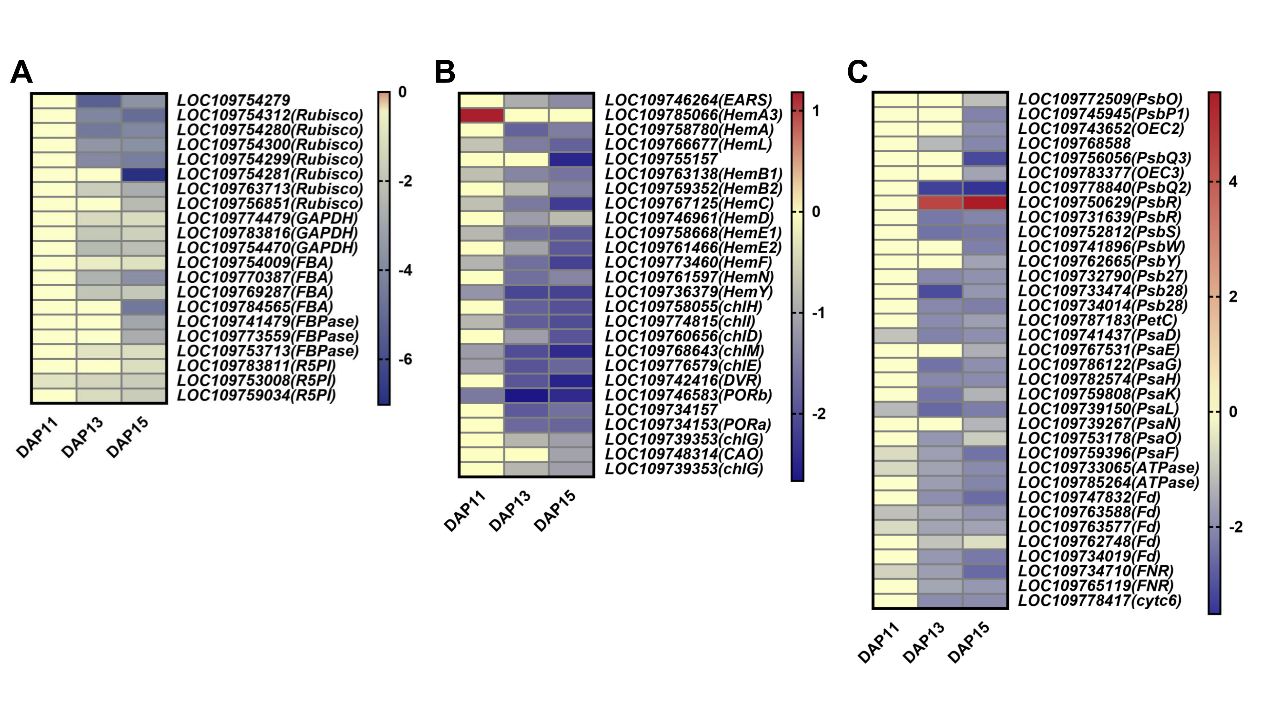
**Figure S3.** The expression levels of DEGs involved in Calvin cycle (A), porphyrin and chlorophyll metabolism (B), and photosynthetic electron transport (C) in *Aegilops tauschii* Coss tiller buds based on log_2_(fold change) with the low plant density treatment as the control at DAP11, DAP13 and DAP15. Each column represents different times, and each row represents one gene.

**Table S1.** Information on the primers used in the quantitative real-time PCR.

| Gene name | Forward primer | Reverse primer |
| --- | --- | --- |
| *LOC109751939* | GCCCAACTCCCCCACCGTCTA | ACTTCCTGTCTTCAATTCTCTGGC |
| *LOC109772458* | CACGAGCACAAGGAGAGTAGC | ACACACACACACACACTTCACAT |
| *LOC109759890* | GCACGCCCAAGGTACGAAATA | GCTCATCTCCTCCACACACACCAA |
| *LOC109778687* | CTAAAGCCACACCGCCACTTG | CCTGCCATCCCACGACCT |
| *LOC109766141* | ACCAGAACAACAACAGCAACC | GCAGGAAAGAAAGTTGAAAGAAAGGC |
| *LOC109763237* | GCGTGGGTAGGAAGGGGTG | ACAGCAACAACAACAAAAAGGGAG |
| *LOC109732743* | TGTCGTCTTCAGTTCCGTTAAGGC | AGGACAAGATAAACAAGAGGAGGAG |
| *AeTubulin* | CCGTTACCTCACCGCCTCT | ATCCACTCCACAAAGTAGGACG |

**Table S2.** Multiple reaction monitoring mode parameters of indole-3-acetic acid, abscisic acid and gibberellin acid 3.

| Compound | Molecular formula | ESI mode | Precursor ion (*m*/*z*) | Quantitative ion (*m*/*z*) | Qualitative ion (*m*/*z*) |
| --- | --- | --- | --- | --- | --- |
| Indole-3-acetic acid | C_10_H_9_NO_2_ | ESI^+^ | 176.0 | 130.0 | 103.0 |
| Abscisic acid | C_15_H_20_O_4_ | ESI^-^ | 263.0 | 153.0 | 219.0 |
| Gibberellin acid 3 | C_19_H_22_O_6_ | ESI^-^ | 345.0 | 143.0 | 239.0 |
